# Tuberous Sclerosis Complex Associated with Papillary Serous Carcinoma of the Peritoneum, Lymphangioleiomyomatosis, and Angiomyolipoma

**DOI:** 10.1155/2011/564260

**Published:** 2011-10-19

**Authors:** Tomihiro Wakamiya, Yasuo Sugita, Mariko Hashiguchi, Tsuyoshi Iwasaka, Osamu Tokunaga

**Affiliations:** ^1^Department of Pathology and Microbiology, Faculty of Medicine, Saga University, Saga City 849-8501, Japan; ^2^Department of Neurosurgery, Faculty of Medicine, Saga University, Saga City 849-8501, Japan; ^3^Department of Pathology, School of Medicine, Kurume University, Japan; ^4^Department of Obstetrics and Gynecology, Faculty of Medicine, Saga University, Saga City 849-8501, Japan

## Abstract

Tuberous sclerosis complex (TSC) is associated with benign and malignant tumors, including lymphangioleiomyomatosis (LAM) and angiomyolipoma (AML). We herein describe the TSC case of a 50-year-old woman having a papillary serous carcinoma of the peritoneum (PSCP), LAM, and AML. On microscopic examination, the PSCP cells showed a cuboidal to columnar shape, proliferated into the papillae, and infiltrated into the peritoneal cavity and anterior thoracic wall. On immunohistochemical evaluation, the tumor cells were positive for epithelial membrane antigen, human epidermal cytokeratins, and progesterone receptor, but negative for calretinin, carcinoembryonic antigen, MCF-7 cell line (Ber-EP4), and estrogen receptor.

## 1. Introduction

Tuberous sclerosis complex (TSC) is associated with benign and malignant tumors [1]. The tumors arise from various sites, including brain, lung, heart, skin, and kidney. However, to our knowledge, papillary serous carcinoma of the peritoneum (PSCP) with lymphangioleiomyomatosis (LAM) and angiomyolipoma (AML) in TSC has never been reported. TSC tends to occur equally among all races and sexes, though AML, LAM, and PSCP occur almost exclusively in women [2]. In this report, we describe an autopsy case of a female patient with TSC, associated with PSCP, LAM, and AML.

## 2. Clinical Summary

**Figure 1:**
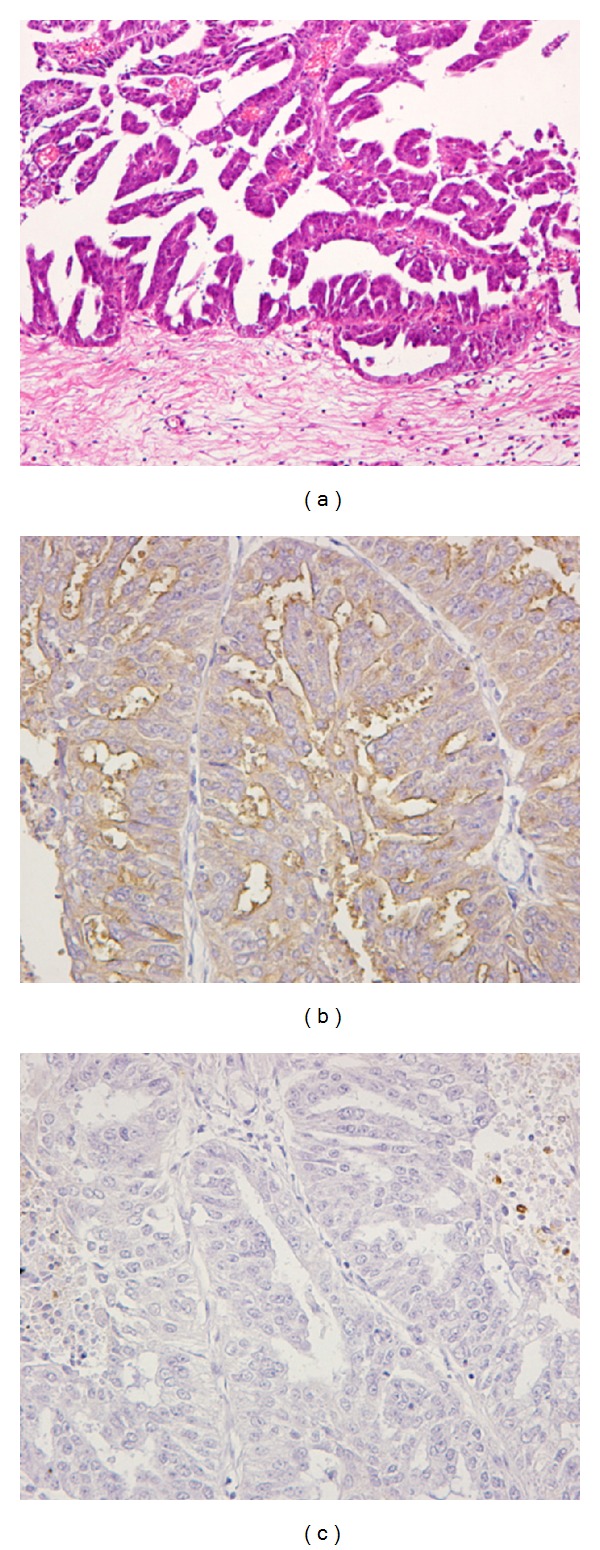
Papillary serous carcinoma of peritoneum (PSCP) on hysterectomy. Cancer cells proliferate and infiltrate in a papillary architecture to the abdominal cavity (a). Epithelial membrane antigen (EMA) is positive (b), but carcinoembryonic antigen (CEA) is negative (c).

A 50-year-old woman visited our hospital, complaining of a lower abdominal distention due to myoma uteri in February, 1998. She has had a leaf-shaped white macule on her back skin (ash leaf macules) since childhood, and she later developed multiple sebaceous adenomas on her face. Although she had no history of either any seizure episodes or mental retardation, she had been diagnosed to have TSC based on subependymal nodules and dot calcification in the bilateral ventricles and AML in both kidneys by computed tomography (CT) scan examination. She had a history of spontaneous pneumothorax at ages 22 and 40. When she underwent a transabdominal hysterectomy and bilateral salpingo-oophorectomy for myoma uteri, diffuse and nodular lesions were found on the serosal surface of the uterus and on the pelvic peritoneum. The histopathologic examination of the peritoneal lesion established the diagnosis of PSCP (Figure 1), and the details are described in a section of pathologic findings. Following surgery, the patient received several cycles of anticancer chemotherapy. The CT scan was performed for an evaluation of anticancer therapy in April, 2003. Multiple masses were found in her pelvic cavity, parietal peritoneum, mesentery, liver, and also in the left thoracic wall and pleura. Both of her kidneys showed tumor masses, and hydronephrosis was noted in the right kidney. Her condition gradually deteriorated and she eventually died of cardiac failure, due to hyperkalemia and renal failure five years after the first operation, hysterectomy.

## 3. Pathologic Findings

**Figure 2:**
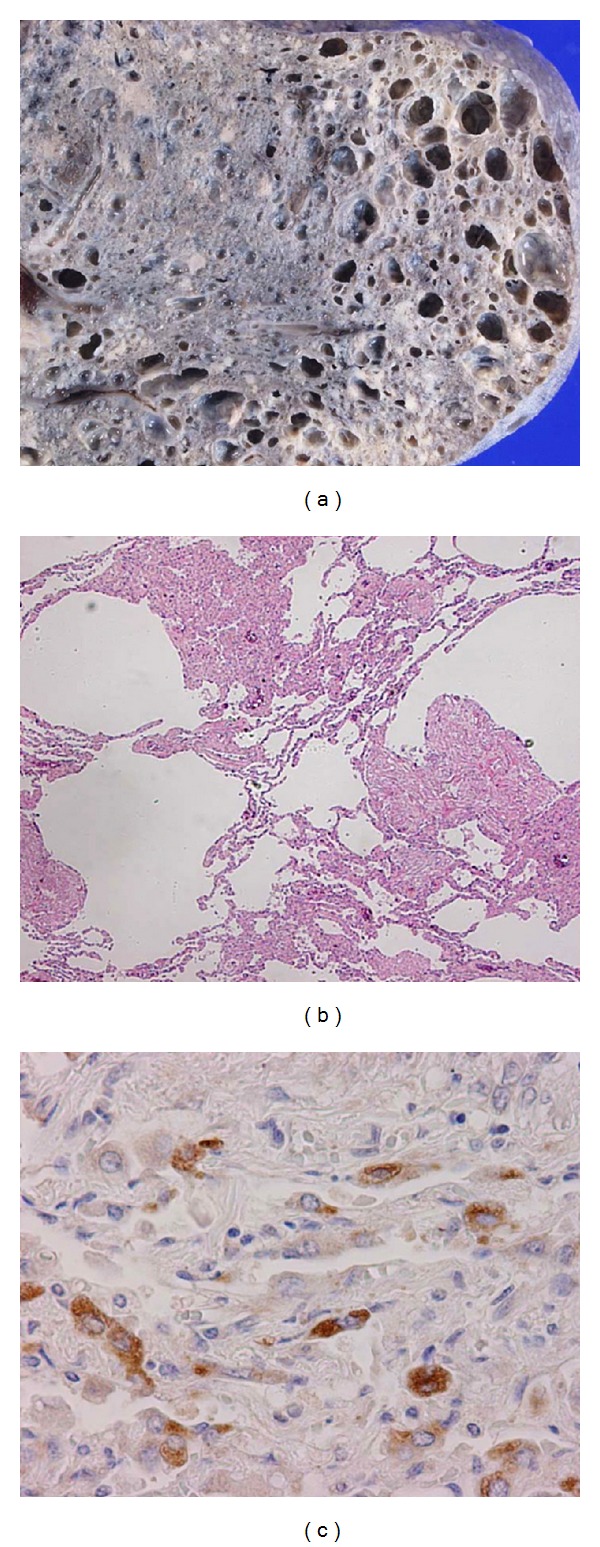
Lymphangioleiomyomatosis (LAM) of lung. In a gross section, multiple cysts are shown in the left lower lung (a). In the HE staining, a dramatic loss of the alveolar septum is associated with an increasing number of cysts. In the cysts, LAM cells are observed to increase in number along the peribronchiole or perilymphatic duct (b). The cytoplasm of the LAM cells is positive for HMB45 (c).

An autopsy was performed. Both lungs showed extensive pleural fibrosis, with adhesion to the thoracic wall, and they also had multilocular cysts throughout (Figure 2(a)). In the abdominal cavity, 2000 mL of bloody ascites was present. The liver, spleen, gastrointestinal tract, gallbladder, and pancreas firmly adhered to each other and formed a single large mass due to either peritonitis carcinomatosa or cancer invasion. The renal corticomedullary boundary was unclear due to multiple tumor nodules, which pushed the renal cortex outerward and spared it in a thin layer. 

The lungs contained multiple thick-walled cysts, which consisted of epithelioid myoid cells and large spindle-shaped cells, along with peribronchiole or peribronchial duct (Figure 2(b)). The cells were immunohistochemically positive for vimentin, desmin, muscle actin (HHF-35), and melanoma-associated antigen (HMB45) (Figure 2(c)), but negative for human progesterone receptor (PgR) and estrogen receptor (ER), supporting the features of LAM. A summary of the immunohistochemical staining of LAM is shown in Table 1. In the center of the nodules, large Type II pneumocytes were present in increased numbers and showed multifocal micronodular pneumocyte hyperplasia.

**Figure 3:**
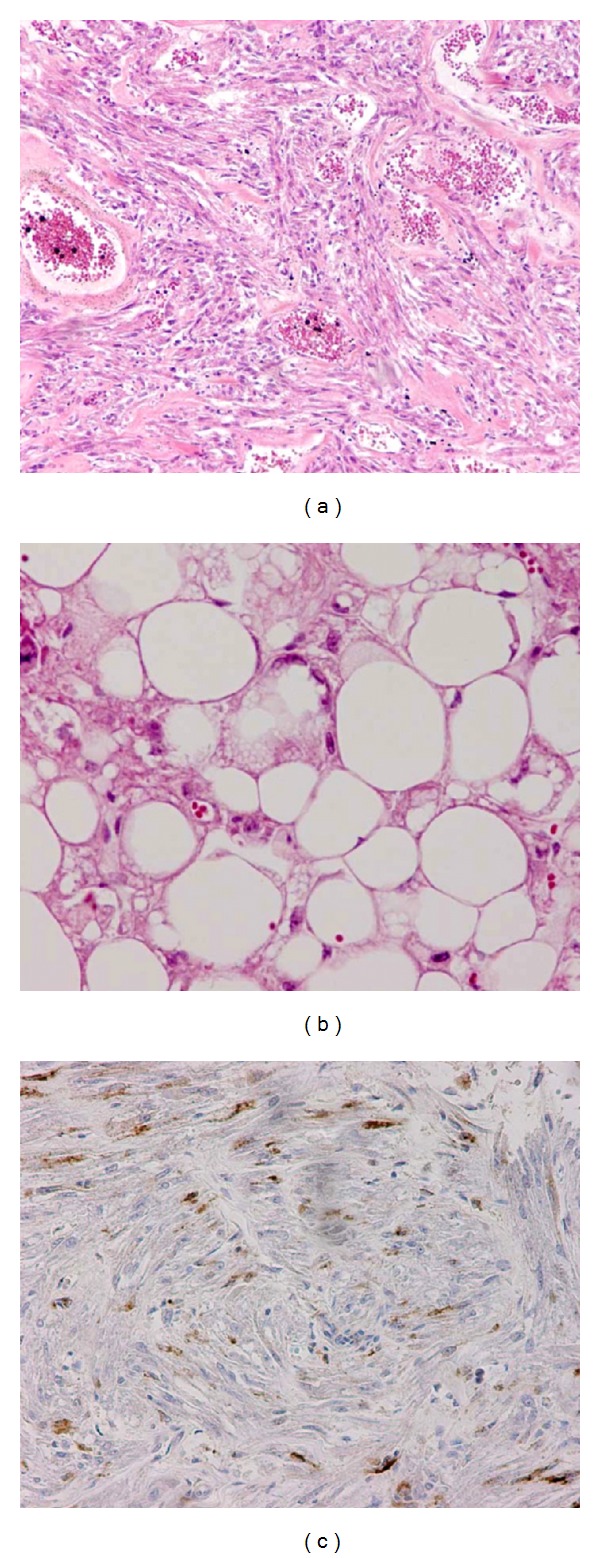
Angiomyolipoma (AML). The proliferation and infiltration of AML cells, including spindle-shaped and epithelioid cells, malformed vessels (a), and fat (b) are demonstrated. HMB-45 is positive (c).

The left kidney contained a large mass comprised of adipocytes, spindle-shaped epithelioid cells, and malformed vessels (Figure 3), consistent with a diagnosis of AML. The cells were negative for PgR and ER.The preserved glomeruli in the thin cortex were congestive, but no glomerular microhamartoma lesion was observed.

**Figure 4:**
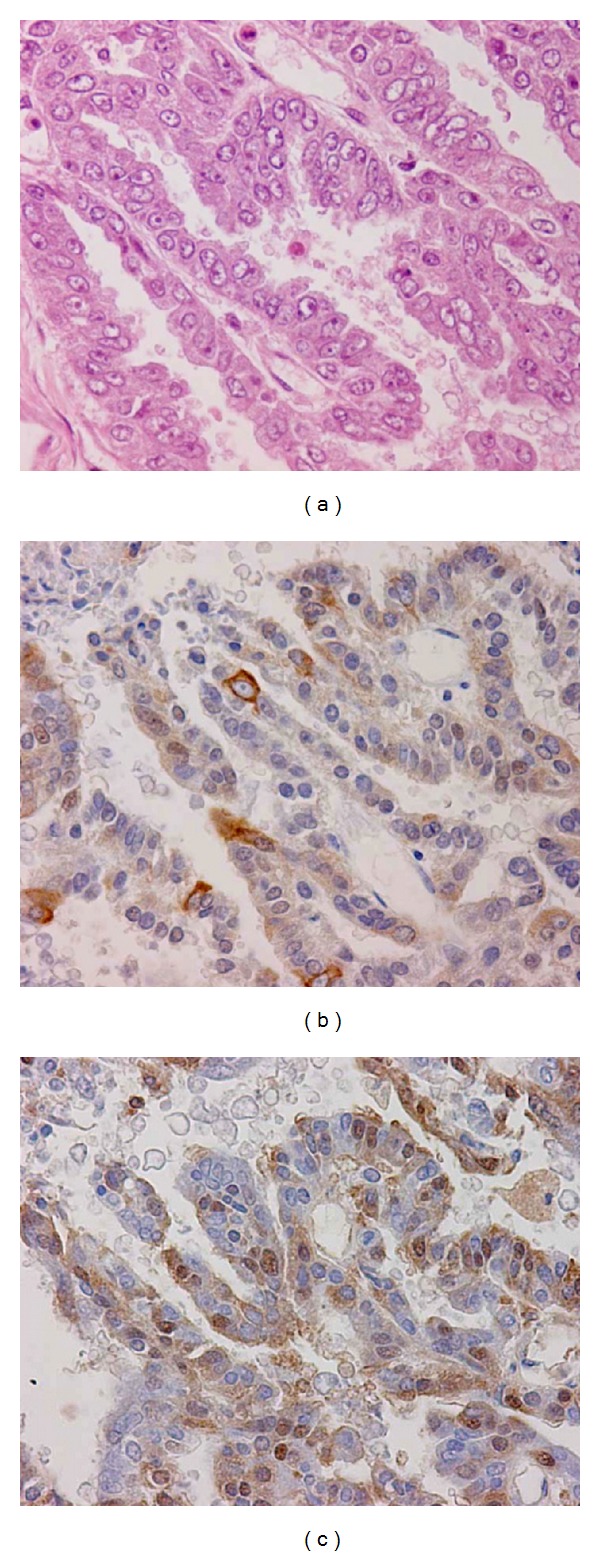
Papillary serous carcinoma of the peritoneum (PSCP). Neoplastic cells which ranged from cuboidal to columnar in shape with polygonal cytoplasm, proliferated and infiltrated in a papillary architecture, to the abdominal cavity and anterior thoracic wall (a). Human epidermal keratins (AE1/AE3) and progesterone receptor (PgR) are positive in (b) and (c), respectively.

The PSCP was same as seen in the previous hysterectomy (Figure 1) and confirmed the diagnosis. The cancer grew in papillary to the abdominal cavity, and the cancer cells were cuboidal to columnar in shape (Figure 4). The invasion of tumor cells extended to the muscle layer of the intestine and to the liver. Psammoma body formation was not seen in this papillary serous carcinoma. On immunohistochemical examination, the tumor was positive for epithelial membrane antigen (EMA), human epidermal keratins (AE1/AE3), and PgR, but negative for calretinin, carcinoembryonic (CEA), ER, and Ber-EP4 (Table 1). The autopsy examination confirmed the previous diagnosis of PSCP. In addition, a small oncocytoma was also incidentally found in the thyroid. The brain examination was not performed because consent for a full autopsy was not obtained from the bereaved family.

## 4. Discussion

PSCP is a rare tumor, which has been described as occurring almost exclusively in women, and the origin of PSCP remains controversial [3]. PSCP morphologically resembles papillary serous carcinoma of the ovary (PSCO) and malignant mesothelioma (MM) [4]. In fact, it is extremely difficult to differentiate between them. Some immunohistochemical markers are of assistance in distinguishing between the carcinomas, particularly calretinin and Ber-EP4 are helpful in distinguishing MM from PSCP and PSCO [5]. 

LAM occurs predominantly in women and develops in approximately 2.3% of patients with TSC [6]. The most common complications are pneumothorax and chylothorax. The median age of the onset of pulmonary symptoms with TSC is 30.4 years, which is childbearing age. The symptoms are worsened by pregnancy, exogenous estrogen, and menstruation. LAM has thus been described as aggravated by estrogen and progesterone treatment [7]. 

AML often occurs in the kidney of patients with TSC, primarily in female patients, and controversy exists regarding the relationship with the sex hormones [8]. In our case, the AML cells were negative for PgR and ER. The relationship between sex hormones and the occurrence of the PSCP remains controversial because PgR and ER are not always expressed in PSCP [9]. In our case, the neoplastic cells of PSCP were positive for PgR, but negative for ER.

TSC is an autosomal dominant disorder associated with the development of malignant and benign tumors including LAM and AML. TSC occurs equally in all races and sexes, though LAM and AML predominantly develop in women. Two genes, *TSC1* and *TSC2,* have been identified and seem to play a specific pathogenic role in TSC [1].

## Figures and Tables

**Table 1:** Summary of immunohistochemical stains in lymphangioleiomyomatosis (LAM) and papillary serous carcinoma of the peritoneum (PSCP).

Antigen	Clone	Source	Dilution	Immunoreactivity	
				LAM	PSCP
Melanoma	HMB-45	Enzo	1 : 40	(+)	
Desmin	D33	Dako	1 : 50	(+)	
Vimentin	V9	Dako	1 : 200	(+)	
Human muscle actin	HHF35	Dako	1 : 200	(+)	
PgR	PR636	Dako	No	(−)	(+)
ER	6F11	Novocastra	1 : 20	(−)	(−)
Human epithelial keratins	AE1/AE3	Chemicon	1 : 200		(+)
EMA	E29	Dako	1 : 100		(+)
Calretinin	Dak Calret1	Dako	1 : 50		(−)
MCF-7 cell line	Ber-EP4	Dako	No		(−)
CEA	II-7	Dako	1 : 100		(−)

Desmin: human desmin. PgR: human progesterone receptor. ER: human estrogen receptor. EMA: epithelial membrane antigen. Calretinin: human calretinin. CEA: carcinoembryonic antigen.
